# Biomechanical analysis of reduction technique for lumbar spondylolisthesis: anterior lever versus posterior lever reduction method

**DOI:** 10.1186/s12891-021-04758-9

**Published:** 2021-10-14

**Authors:** Yu-Tsung Lin, Kuo-Chih Su, Kun-Hui Chen, Chien-Chou Pan, Cheng-Min Shih, Cheng-Hung Lee

**Affiliations:** 1 Department of Orthopedics, Taichung Veterans General Hospital, Taichung, Taiwan; 2 Department of Medical Research, Taichung Veterans General Hospital, Taichung, Taiwan; 3 Department of Biomedical Engineering, Hungkuang University, Taichung, Taiwan; 4 Department of Chemical and Materials Engineering, Tunghai University, Taichung, Taiwan; 5 Department of Computer Science and Information Engineering, Providence University, Taichung, Taiwan; 6 National Chung Hsing University, Taichung, Taiwan; 7 Department of Rehabilitation Science, Jenteh Junior College of Medicine, Nursing, and Management, Miaoli County, Taiwan; 8 Department of Physical Therapy, Hungkuang University, Taichung, Taiwan; 9 Department of Food Science and Technology, Hung Kuang University, Taichung, Taiwan

**Keywords:** Anterior lever reduction, Posterior lever reduction, Lumbar spondylolisthesis, Biomechanical study

## Abstract

**Background:**

Reduction of lumbar spondylolisthesis during spinal fusion surgery is important for improving the fusion rate and restoring the sagittal alignment. Despite the variety of reduction methods, the fundamental mechanics of lumbar spondylolisthesis reduction remain unclear. This study aimed to investigate the biomechanical behavior while performing spondylolisthesis reduction with the anterior and posterior lever reduction method.

**Methods:**

We developed an L4–L5 spondylolisthesis model using sawbones. Two spine surgeons performed the simulated reduction with a customized Cobb elevator. The following data were collected: the torque and angular motion of Cobb, displacement of vertebral bodies, change of lordotic angle between L4 and L5, total axial force and torque applied on the model, and force received by adjacent disc.

**Results:**

Less torque value (116 N-cm vs. 155 N-cm) and greater angular motion (53^o^ vs. 38^o^) of Cobb elevator were observed in anterior lever reduction. Moreover, the total axial force received by the entire model was greater in the posterior lever method than that in the anterior lever method (40.8 N vs. 16.38 N). Besides, the displacement of both vertebral bodies was greater in the anterior lever method.

**Conclusions:**

The anterior lever reduction is a more effort-saving method than the posterior lever reduction method. The existing evidence supports the biomechanical advantage of the anterior reduction method, which might be one of the contributing factors to successfully treating high-grade lumbar spondylolisthesis with short-segment instrumentation.

## Background

Lumbar spondylolisthesis is characterized by the forward slippage of one vertebra over the one beneath it. Surgical intervention is generally recommended for patients with high-grade spondylolisthesis after the failure of conservative treatment [1]. The mainstay of surgery for spondylolisthesis is instrumented spinal fusion with or without reduction. Although the superiority of clinical outcomes following instrumented fusion with reduction versus instrumented fusion in situ is highly debated [2–5], reduction of the spondylolisthesis may enhance the rate of fusion by increasing the bony contact and the area in compression, reducing the stress across the fusion mass [6]. According to a systemic review comparing fusion in situ with fusion after reduction, fusion after reduction was found to decrease slippage percentage and improve the fusion rate of spondylolisthesis [5]. Specifically, in patients with high-grade spondylolisthesis with abnormal posture, reduction and realignment procedures should be performed to restore the global spinopelvic balance [5, 7, 8].

The most commonly used approach for spinal fusion is the posterior approach. The posterior approach can be used in instrumented interbody fusion techniques, such as posterior lumbar interbody fusion (PLIF) or transforaminal lumbar interbody fusion (TLIF), which leads to a satisfactory outcome in patients with spondylolisthesis [9]. The anterior approach of the lumbar spine, which can be used in instrumented interbody fusion technique of anterior interbody fusion (ALIF), can facilitate access to the intervertebral space without passing through the spinal canal with retraction of the nerve roots and cauda equina, reducing the potential risk of nerve injury and dural tear [10].

Several studies have compared the clinical outcomes of anterior and posterior spinal fusion as a treatment for lumbar spondylolisthesis. Tye et al. have compared ALIF and TLIF in patients with isthmic spondylolisthesis. Their results revealed that the functional score improved significantly for both groups 1 year after the operation. However, the EuroQol-5D (EQ-5D) scores improved significantly more in patients in the ALIF group than those in the TLIF group [11]. Besides, Min et al. have also found that in patients who underwent L4–L5 fusion surgery for spondylolisthesis, ALIF is more advantageous in preventing the development of adjacent segment disease (ASD) compared with PLIF [12].

Among the reduction techniques, the lever reduction technique through the anterior approach was first described by Bradford et al. [13]. In this procedure, the tip of a Hohmann retractor was positioned at the posterior rim of the upper endplate of the lower vertebrae, which was then used as a fulcrum to reduce the slippage of the spondylolisthesis. In contrast, Kong et al. [14] have also developed a lever reduction technique that can achieve reduction with a lever repositioner via the posterior approach.

Although the anterior spinal fusion appears to have several clinical advantages over posterior spinal fusion, previous biomechanical studies mostly focused on the stability and alignment changes of the spinal fusion structure after the procedure [15–18]. No studies have so far investigated behavior while performing the spondylolisthesis reduction and the difference between the anterior and posterior reduction techniques.

This study aimed to investigate the biomechanical behavior while performing reduction maneuvers of spondylolisthesis using the anterior and posterior lever reduction methods. This information can provide clinical physicians the basic mechanics of different reduction techniques in spondylolisthesis surgery.

## Methods

### Establishment of spondylolisthesis model

**Fig. 1:**
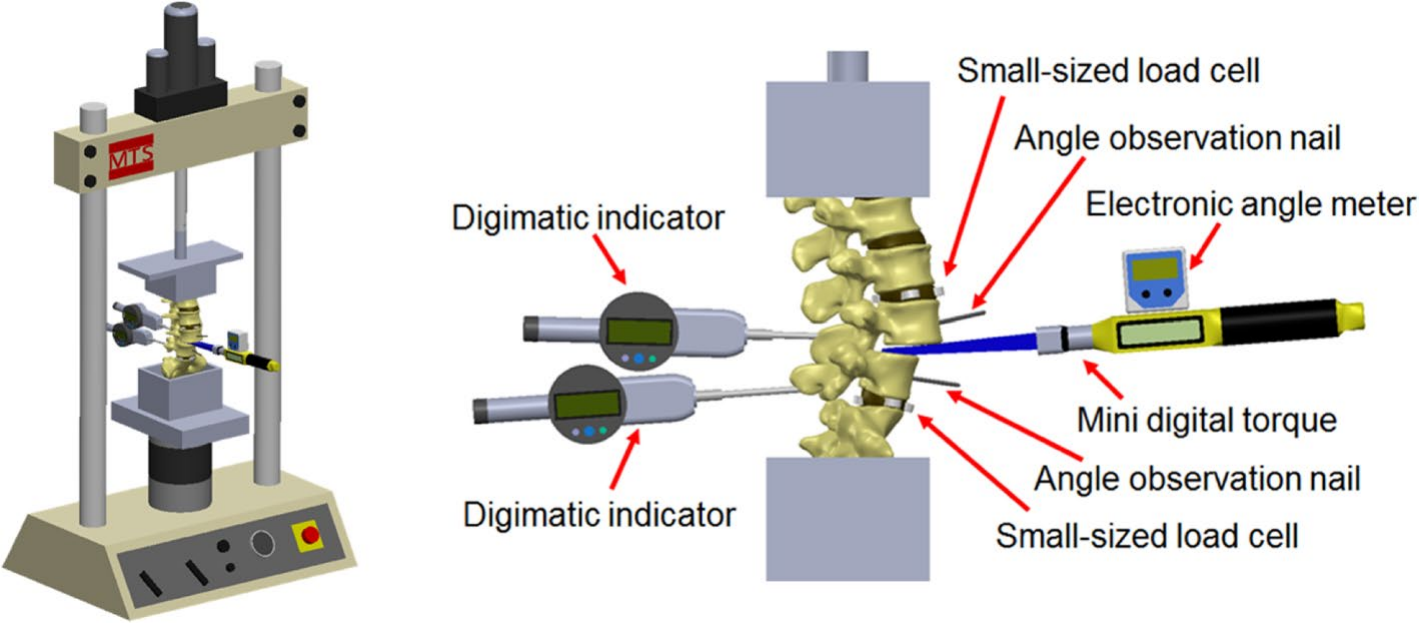
The spondylolisthesis model was mounted on the mechanical testing system tensile testing machine. Mechanical instruments were set up to measure different parameters during spondylolisthesis reduction

Twenty lumbar spine sawbone models (lumbar vertebrae, model 1352, Pacific Research Laboratories, Vashon, WA, USA) were used as experimental models to examine the effects of anterior lever and posterior lever reduction methods. The intervertebral disc and anterior and posterior longitudinal ligaments of L4–L5 segment were resected. The L4 vertebrae were positioned at the anterior third of the L5 vertebrae, which simulated Meyerding grade 3 spondylolistheses [19]. A customized clamp was subsequently used to set up the spine sawbone model on a mechanical testing system (MTS 858 Mini Bionix Testing Machine; MTS Systems Corp., Eden Prairie, MN) (Fig. 1).

### Reduction procedure

**Fig. 2:**
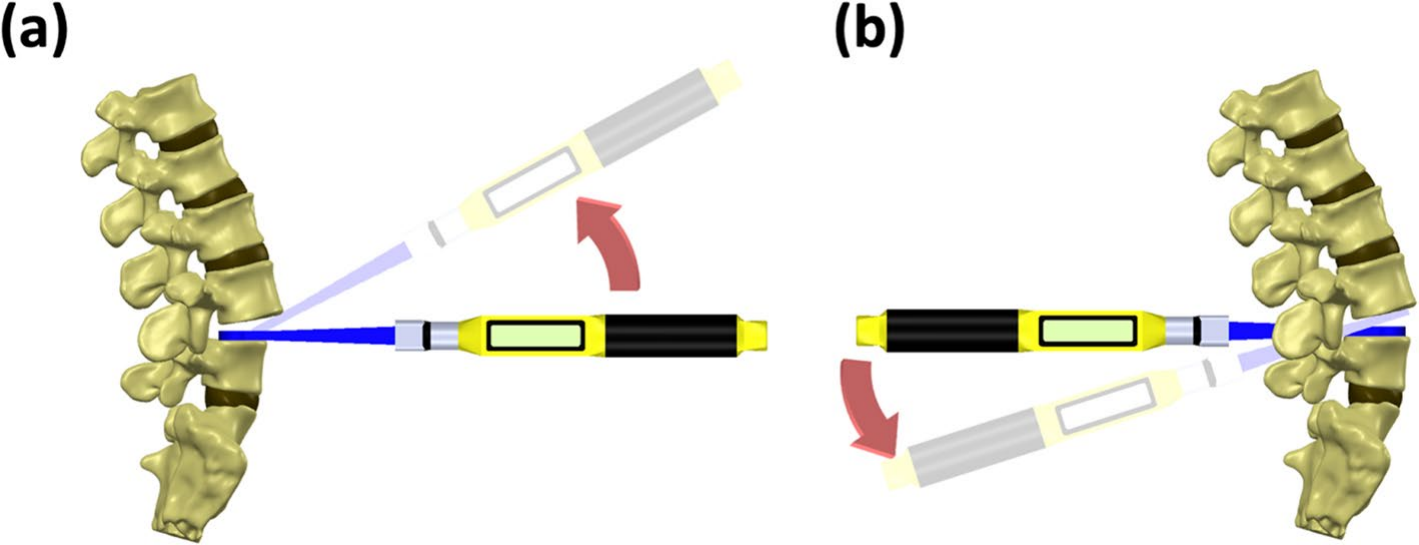
Illustration of the reduction process. **a** anterior lever reduction; **b** posterior lever reduction

The procedures in the present study were performed by two experienced spine surgeons. Each of them performed 25 anterior lever reductions in five sawbones and 25 posterior lever reductions in another five sawbones. During anterior lever reduction, the surgeon applied a Cobb elevator to the intervertebral body space anteriorly. The tip of the Cobb was placed at the posterior rim of the L5 upper endplate, which served as a fulcrum (Fig. 2a). Subsequently, a force was applied to pry the slipped vertebrae until reduction was achieved based on the surgeon’s judgment.

During posterior lever reduction, a Cobb elevator was applied through the interbody space either from the left or the right side. The tip of the Cobb was placed at the anterior rim of the L4 lower endplate, and the L5 vertebrae served as a lever fulcrum when performing lever reduction (Fig. 2b). Force was subsequently applied to pry the slipped vertebrae backward until reduction was achieved based on the surgeon’s judgment.

### Setting of the mechanical testing system

The spondylolisthesis model was set up on the MTS tensile testing machine. Mechanical instruments were used to measure the change in mechanics of the lumbar vertebrae during reduction (Fig. 1). An electronic angle meter and mini digital torque wrench were installed on the customized Cobb elevator to measure the angular motion and torque of the Cobb elevator while performing reduction maneuver. The electronic angle meter sensed the angular change of Cobb. The mini digital torque wrench (WM-106-1, Asmith Manufacturing Company, Taiwan) detected the torque, with the range of measurement between 30 and 600 N-cm.

To observe the movement of vertebral bodies during spondylolisthesis reduction, two digimatic indicators (Mitutoyo digimatic indicator, Type ID-C1050MXB, Mitutoyo Manufacturing Co. Ltd., Tokyo, Japan) were set up at the spinous processes of L4 and L5. The indicators were used to measure forward and backward displacement of the reduced vertebral body, with the range of measurement up to 50.8 mm. Furthermore, two angle observation nails were inserted into the center of the anterior vertebral bodies of L4 and L5, which were used to measure the change of lordotic angle between the L4 and L5 vertebral bodies after performing the reduction.

The lumbar spine sawbone model was set up on the MTS machine (MTS 858 Mini Bionix) so that the cells in the MTS could measure both the total axial force and total torque applied on the model. These two forces represented the resultant force received by the entire lumbar spine while a surgeon performed the reduction.

**Fig. 3:**
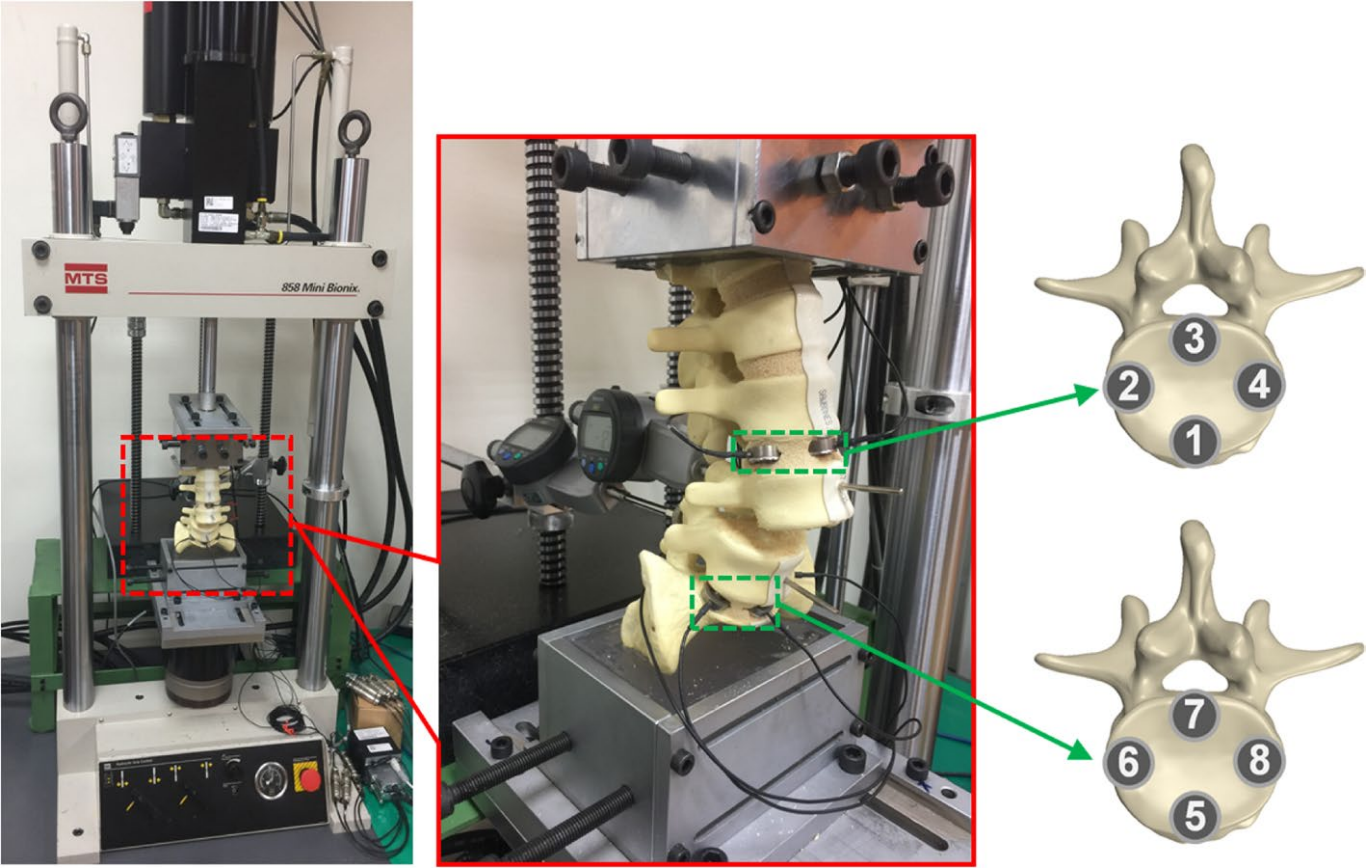
Small-sized compression load cells were inserted at the adjacent intervertebral disc. No.1-No.4 load cells were placed at L3/4 intervertebral disc. No.5–8 load cells were placed at L5/S1 intervertebral disc. The model was then set up on the mechanical testing system machine

To observe the force created over the adjacent intervertebral disc during spondylolisthesis reduction, eight small-sized compression load cells (LMB-A-2KN, Kyowa Electronic Instruments Co. Ltd., Tokyo) were used to measure the force distribution in intervertebral discs near L4 and L5. The maximum force that could be measured was 2 kN. Small-sized load cells were inserted at the anterior, posterior, left, and right positions of the intervertebral discs at the L3–L4 and L5–S1 segments. The cells inserted in L3–L4 were numbered 1–4, whereas those inserted into the L5–S1 intervertebral disc were numbered 5–8 (Fig. 3).

Differences between the measured data of the two methods were tested using Student’s t-test. The statistical analysis was performed using Statistical Package for Social sciences version 22.0 (SPSS Inc., Armonk, NY). A *p*-value of < 0.05 was considered statistically significant.

## Results

**Fig. 4:**
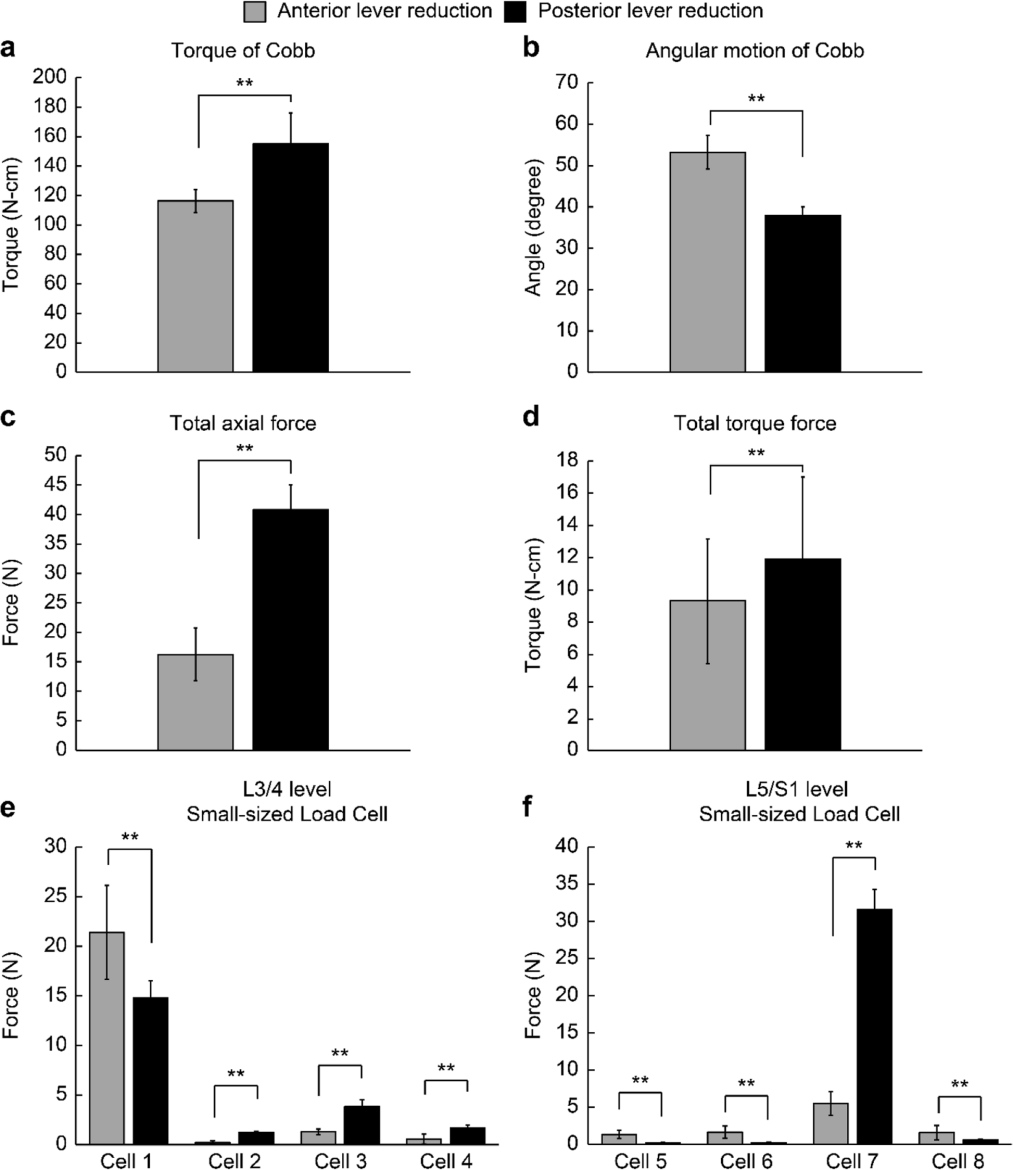
**a** Torque value of Cobb recorded by mini digital torque wrench; **b** Cobb angular motion recorded by an electric angle meter; **c** Total axial force recorded by an MTS machine; **d** Torque value recorded by a mechanical testing system machine; **e** Force distribution at L3/L4 level; and **f** Force distribution at L5/S1 level; *p <  0.05, **p <  0.01

Observation of the mini digital torque wrench on Cobb elevator revealed that anterior lever reduction led to significantly lesser torque on Cobb (116 N-cm vs. 155 N-cm, *p* <  0.001, Table 1, Fig. 4a). Moreover, the electronic angle meter on Cobb elevator showed that anterior lever reduction had greater angular motion compared with posterior lever reduction (53^o^ vs. 38^o^, *p* <  0.001, Table 1, Fig. 4b).

**Table 1:** Recorded parameters

	Anterior lever reduction	Posterior lever reduction	***p*** Value
Torque of Cobb (N-cm)	116 ± 7.7	155 ± 21	< 0.001**
Angular motion of Cobb (degree)	53.22 ± 4.1	37.96° ± 2	< 0.001**
Displacement of vertebral body (mm)			
L4 (backward displacement)	7.35 ± 1.52	6.74 ± 0.97	0.02*
L5 (forward displacement)	1.57 ± 0.25	0.87 ± 0.25	< 0.001**
Change of lordotic angle (Δ degree)	−2.39 ± 1.52	− 4.88 ± 1.94	< 0.001**
Total axial force of the model (N)	16.38 ± 4.51	40.8 ± 4.54	< 0.001**
Total torque of the model (N-cm)	9 ± 3.9	12 ± 5.1	0.003**

Student’s t-test, *p <  0.05, **p <  0.01; Mean values are presented ± SD

The digimatic indicators showed that L4 would undergo backward displacement after reduction (Table 1), whereas L5 would be displaced slightly forward. Displacement of L4 and L5 vertebral bodies were significantly higher in the anterior lever method than in the posterior (Table 1). The observation nails revealed that the posterior lever reduction decreased the lordotic angle more than the anterior lever reduction, gaining significant difference (− 2.4° and − 4.9°, *p* <  0.001, Table 1).

Posterior lever reduction added a greater total axial force to the lumbar spine model (40.8 N vs. 16.38 N, *p* <  0.001, Fig. 4c). Moreover, posterior lever reduction also resulted in greater torque to the model. However, the torque values were small in both methods (12 N-cm vs. 9 N-cm, *p* = 0.003, Fig. 4d).

Among the four load cells at L3–L4 disc level (Table 2, Fig. 4e), cell no. 1, which was located at the anterior position of this level, experienced the highest force in both methods, which was higher in the anterior lever reduction compared with the posterior lever reduction method (21.4 N vs. 14.78 N, *p* <  0.001, Table 2). Among the four cells at L5–S1 level (Table 2, Fig. 4f), cell no. 7, which was located at the posterior position at this level, experienced the highest force in both methods, which was higher in the posterior lever reduction compared with the anterior lever reduction (31.54 N vs. 5.5 N, *p* <  0.001, Table 2).

**Table 2:** Force recorded by small-sized load cell (N) at adjacent disc

Small-sized load cell	Anterior lever reduction	Posterior lever reduction	***p*** Value
L3/L4 intervertebral level			
Cell No. 1	21.4 ± 4.74	14.78 ± 1.74	< 0.001**
Cell No. 2	0.22 ± 0.21	1.24 ± 0.11	< 0.001**
Cell No. 3	1.3 ± 0.28	3.82 ± 0.71	< 0.001**
Cell No. 4	0.57 ± 0.51	1.69 ± 0.28	< 0.001**
L5/S1 intervertebral level			
Cell No. 5	1.34 ± 0.57	0.16 ± 0.12	< 0.001**
Cell No. 6	1.65 ± 0.81	0.23 ± 0.1	< 0.001**
Cell No. 7	5.5 ± 1.62	31.54 ± 2.75	< 0.001**
Cell No. 8	1.58 ± 0.97	0.6 ± 0.13	< 0.001**

Student’s t-test, *p < 0.05, **p < 0.01; Mean values are presented ± SD

## Discussion

The reduction of spondylolisthesis plays an important role in enhancing fusion rate while preventing slippage progression after instrumented spinal fusion, especially in high-grade spondylolisthesis patients [5]. Clinically, in patients with low-grade spondylolisthesis, either anterior or posterior reduction followed by mono-segment instrumentation provides excellent fusion rate and satisfactory functional outcome [9, 20, 21]. Several studies have compared the outcome between anterior and posterior spinal fusion in this population. Tye et al. found that both ALIF and TLIF could significantly improve the functional score 1 year after the operation. However, ALIF reportedly results in significantly greater improvement in EQ-5D scores than TLIF [11]. Furthermore, Min et al. have also reported that ALIF is more advantageous in preventing the development of ASD compared with PLIF [12].

In patients with high-grade spondylolisthesis, the treatment of anterior reduction followed by single-level spinal fusion was first described by Bradford et al. [13]. Later, David et al. [22] found that single-level ALIF, followed by mono-segment posterior instrumentation, provides excellent outcomes in this patient population. All the patients included in their study had achieved bony fusion. Moreover, complete reduction of slippage had been observed in 87.5% of these patients at the 17-month follow-up. Furthermore, in a preliminary report conducted by Tu et al., anterior cantilever reduction procedure followed by ALIF and posterior mono-segment instrumented fusion could achieve a high fusion rate while correcting lumbosacral angle [23]. On the contrary, Lengert et al. [24] found that patients who underwent posterior reduction with mono-segment posterior instrumentation experienced loss of reduction 1 year after the operation. Therefore, posterior L4–S1 fusion provided better outcomes than single-level L5–S1 fusion in patients with high-grade L5/S1 spondylolisthesis.

Our findings suggest that the anterior lever reduction method can achieve spondylolisthesis reduction in an effort-saving manner without adding excessive force over the entire lumbar spine. This result supports the existing evidence that the anterior lever reduction technique has a mechanical advantage over the posterior lever reduction technique, which might be one of the contributing factors to treat high-grade lumbar spondylolisthesis with short-segment instrumentation successfully.

**Fig. 5:**
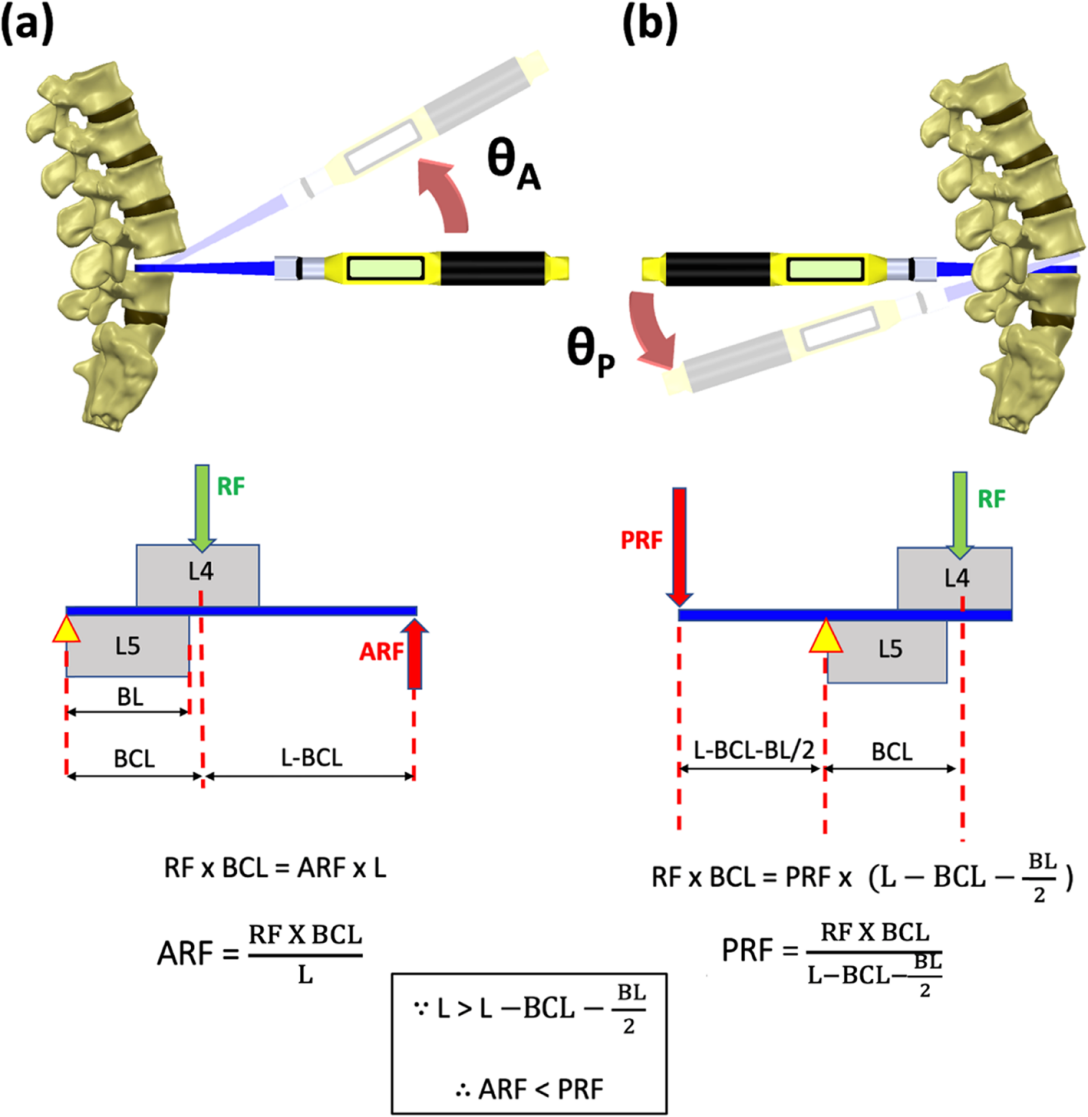
Mechanical free body diagrams of both reduction methods. The lever arm of anterior lever reduction is greater than the posterior reduction method(L > L-BCL-(BL)/2); therefore, the force required to achieve reduction is lesser (ARF < PRF). **a** Anterior lever reduction involves a class 2 lever; **b** Posterior lever reduction involves a class 1 lever. ARF, anterior reduction force; PRF, posterior reduction force; RF, resistance force; L: Cobb length; BL: body length; BCL: L4 body center length

From a mechanical viewpoint, the anterior lever reduction method involves a class 2 lever, whereas the posterior lever reduction method involves a class 1 lever. Because the length of the lever arm is greater (Fig. 5, L > L-BCL-(BL)/2), anterior lever reduction requires lesser force to achieve reduction than posterior lever reduction (Fig. 5, ARF < PRF), which finally leads to lesser torque on Cobb and lesser resultant force to the entire lumbar spine. Figure 5 presents the mechanical free body diagrams to analyze the force applied by the surgeon during lever reduction.

Anterior approach of the spine has become an effective and popular alternative for achieving lumbar fusion. The anterior and middle column provides 80% of the weight-bearing load of the spinal column, and the anterior approach offers efficient and direct access to this area [25]. In the present study, we found that the angular motion of Cobb during reduction was greater while performing the anterior lever reduction method compared with the posterior lever reduction method. This is because the anterior approach of the spine was not hindered by the complex structure of the posterior spine. Therefore, a surgeon can achieve reduction with Cobb through an ideal angle (Fig. 5, θ_A_ > θ_P_).

The backward displacement of L4 and forward displacement of L5 vertebral bodies were both greater using the anterior lever method. This result suggested that anterior reduction can achieve a better degree of slip reduction by applying lesser force to the lumbar spine, proving that the anterior reduction is biomechanically more efficient than posterior reduction. Despite this advantage, in patient with high-grade spondylolisthesis, partial reduction may be safer than complete reduction. According to Bradford et al. [26], the risk of iatrogenic neurologic injury correlates with the degree of reduction obtained. In previous literature, partial reduction of high-grade spondylolisthesis seemed to provide a satisfactory outcome with a low incidence of nerve traction injury after the procedure [23]. Therefore, our results suggest that both methods can successfully achieve reduction. Meanwhile, the risk of nerve root stretch injury among both methods may be similar if the goal of slippage reduction is the same. Regardless of the reduction method, the reduction process will add pressure on the adjacent segments. The data from the small-sized load cells revealed that the anterior reduction method might increase pressure over the upper adjacent disc (Cell No.1 at L3/4 level). In contrast, the posterior reduction may increase pressure over the lower adjacent disc (Cell No.7 at L5/S1 level).

Our study has a few limitations. The L4-L5-S1 segments contribute to two-thirds of the lordotic angle of the lumbar spine. The intervertebral disc, anterior and posterior longitudinal ligaments were removed to establish a high-grade spondylolisthesis model. This created hyperlordosis of the lumbar spine due to vertebral body slippage without ligament constraint. However, a normal lordosis angle can be achieved after a reduction maneuver, which decreases lordotic angle in both methods. This is somewhat different from the clinical scenario [23]. Second, stretching and subsequent straining of the nerve root can cause iatrogenic neurologic injury during spondylolisthesis reduction, which should be monitored during the operation. However, this could not be simulated in our model. Third, although the segment most commonly affected by degenerative spondylolisthesis is L4-L5, most cases of spondylolisthesis occur at L5–S1. However, the spondylolisthesis model simulated in this study was set at L4–L5 because of the difficulty in setting mechanical instruments at the L5–S1 segment in a sawbone model. Finally, although the force over the adjacent disc was observed in the spondylolisthesis model, the force applied over vertebral endplates was not measured, which may be important, especially in osteoporosis patients because of the vulnerability of endplate that can be violated during the reduction procedure.

Although the models used in the present study differed relatively from the clinical situation, the trends of biomechanical results remained similar. We also established a new lumbar spine mechanical measurement system by employing an MTS machine to collect more data than previous experiments.

## Conclusions

The anterior lever reduction is a more effort-saving method than the posterior lever reduction method. The existing evidence supports the biomechanical advantage of the anterior reduction method, which might be one of the contributing factors to successfully treating high-grade lumbar spondylolisthesis with short-segment instrumentation.

## Data Availability

Data and materials are available from the corresponding author under at a reasonable request.

## Abbreviations

ALIF: Anterior lumbar interbody fusion
ASD: Adjacent segment disease
MTS: Mechanical testing system
PLIF: Posterior lumbar interbody fusion
TLIF: Transforaminal lumbar interbody
